# Understanding of MYB Transcription Factors Involved in Glucosinolate Biosynthesis in Brassicaceae

**DOI:** 10.3390/molecules22091549

**Published:** 2017-09-14

**Authors:** Mi-Suk Seo, Jung Sun Kim

**Affiliations:** Genomics Division, Department of Agricultural Bio-Resources, National Institute of Agricultural Sciences, Rural Development Administration, Wansan-gu, Jeonju 54874, Korea; sms1030@korea.kr

**Keywords:** glucosinolate, MYB transcription factor, polyploid, R2R3 MYB DNA-binding domain, *Brassica*

## Abstract

Glucosinolates (GSLs) are widely known secondary metabolites that have anticarcinogenic and antioxidative activities in humans and defense roles in plants of the Brassicaceae family. Some R2R3-type MYB (myeloblastosis) transcription factors (TFs) control GSL biosynthesis in *Arabidopsis*. However, studies on the MYB TFs involved in GSL biosynthesis in *Brassica* species are limited because of the complexity of the genome, which includes an increased number of paralog genes as a result of genome duplication. The recent completion of the genome sequencing of the *Brassica* species permits the identification of MYB TFs involved in GSL biosynthesis by comparative genome analysis with *A. thaliana*. In this review, we describe various findings on the regulation of GSL biosynthesis in Brassicaceae. Furthermore, we identify 63 orthologous copies corresponding to five MYB TFs from *Arabidopsis*, except MYB76 in *Brassica* species. Fifty-five MYB TFs from the *Brassica* species possess a conserved amino acid sequence in their R2R3 MYB DNA-binding domain, and share close evolutionary relationships. Our analysis will provide useful information on the 55 MYB TFs involved in the regulation of GSL biosynthesis in *Brassica* species, which have a polyploid genome.

## 1. Introduction 

Plants produce various secondary metabolites that are involved in traits such as taste, color, and scent, and have roles in plant defense against environmental changes or stress unrelated to the primary functions of plants, such as development, reproduction, and photosynthesis [1]. More than 200,000 secondary metabolites are known in plants, and humans have utilized plants for their various benefits (natural medicines, flavors, insecticides, and industrial materials) obtained through the wide chemical diversity of metabolites [2]. Recent studies have reported that cruciferous (Brassicaceae) vegetables are rich in secondary metabolites, including carotenoids, flavonoids, anthocyanins, and glucosinolates (GSLs) [3]. GSLs are derived from amino acids and sugars and are one of the largest known groups of secondary metabolites in the Brassicaceae family. GSLs and their breakdown products have recently attracted attention owing to their various beneficial roles, such as anticarcinogenic and antioxidative activities in humans, and defense against pests and pathogens in plants [2,4]. Therefore, understanding of the GSL biosynthesis pathway will increase their nutritional value and provide agriculturally useful information related to the defense mechanisms in Brassicaceae plants.

The Brassicaceae family consists of 338 genera and about 3700 species, including *Arabidopsis thaliana*, which is widely studied as a model plant with a small genome, and many plants with agronomic importance such as vegetables, fodder, oil crops [5]. The agricultural and nutritional properties of Brassicaceae plants have resulted in their extensive cultivation. The genus *Brassica*, which is a member of Brassicaceae, contains the vegetable species, *Brassica rapa* (A genome, Chinese cabbage, pak choi), *Brassica oleracea* (C genome, kale, broccoli, cauliflower), *Brassica nigra* (B genome, black mustard), and *Brassica napus* (A and C genome, canola). These *Brassica* species are known as the ’triangle of U’ regarding the genome evolution and the relationships between members of the *Brassica* genus, including three diploid and three allopolyploid genomes. The genomes of these *Brassica* species contain triplicated whole genomes of *A. thaliana* [6]. Thus, a comprehensive approach including *A. thaliana* and *Brassica* species will provide useful information on polyploidy-related genome evolution. Recently, the genome sequences of *B. rapa* and *B. oleracea*, with diploid genomes, and *B. juncea* and *B. napus*, with allopolyploid genomes, have been published and used to explain the evolutionary relationships between *Brassica* species [7,8,9,10]. The completion of the polyploid genome sequence of the *Brassica* species permits the identification of various gene families of secondary metabolites by comparative genome analysis with *A. thaliana*.

Recent studies have shown that genome polyploidy leads to the amplification or redundancy of genes, and duplicated genes with functional diversity distinct from their ancestral function [11]. One of the representative functional genes in late flowering, *FLC (FLOWERING LOCUS C)* have five copies in the *B. rapa* genome; these five copies are the result of genome triplication (*BrFLC1*, *2*, *3*) and additive recent duplication (*BrFLC3a*, *b*) as well as gene introgression (*BrFLC5*) [12]. In our previous study, *BrFLCs* showed different expression levels in tissue (leaf and root) and in unvernalized and vernalized *B. rapa* [13]. Such variation in the copy number of genes could play a major role in regulatory processes in the *Brassica* genus. A genome-wide comparison of genes related to GSL biosynthesis suggested that genome duplication resulted in the expansion of GSLs-related genes in the *Brassica* genome [10,14,15]. Furthermore, functional analysis of these genes has demonstrated that GSL biosynthesis in *Brassica* species is controlled by more regulators than in *Arabidopsis*, because of their polyploid genomes [16,17]. This indicates that GSL biosynthesis of *Brassica* species has a regulatory network distinct from *Arabidopsis*. Recently, some MYB TFs were described as the regulatory factors directly or indirectly activating GSL pathway genes [18,19,20]. Studies have used various molecular biological approaches to investigate GSL biosynthesis in *A. thaliana* and *Brassica* species [16,17,18]. Some R2R3 MYB transcription factors (TFs) with two MYB repeats (R2R3) control GSL biosynthesis. In particularly, MYB28, MYB29, and MYB76 are positive regulators of aliphatic GSL synthesis in *Arabidopsis* [19]. Furthermore, MYB34, MYB51, and MYB122 have been shown to regulate indolic GSL synthesis [21].

In this review, we describe these various findings to provide an integrative understanding of divergence and a functional diversification of the MYB TFs involved in GSL biosynthesis following genome duplication in *A. thaliana* and *Brassica* species.

## 2. Metabolic and Regulatory Pathways of GSL Biosynthesis

### 2.1. Metabolic Pathway of GSLs

**Figure 1:**
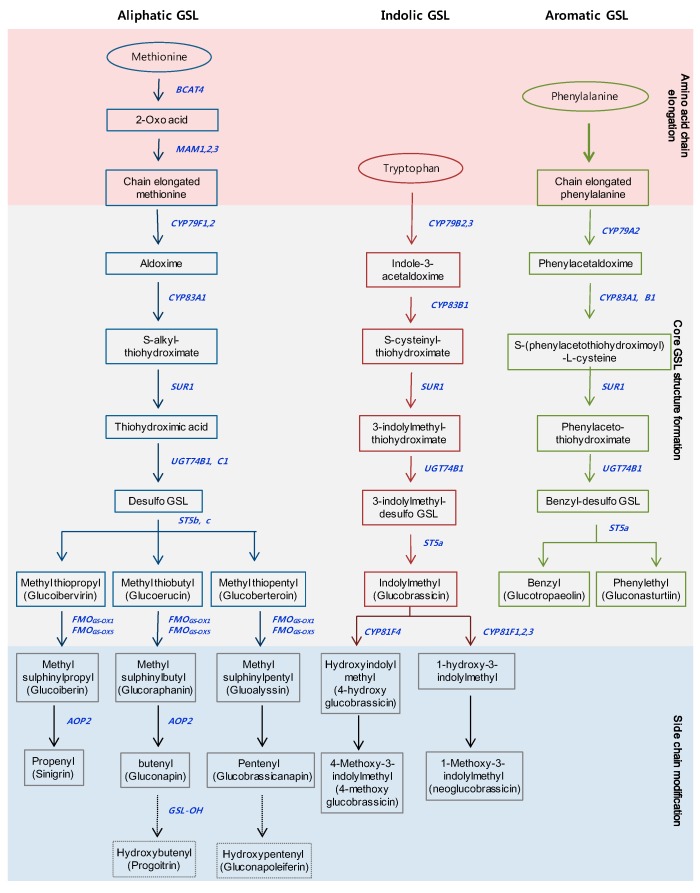
Three major biosynthesis pathways of the glucosinolates in Brassicaceae. Outline of the pathways which can be divided into three parts; (1) amino acid chain elongation; (2) formation of the core structure of GSLs; (3) side chain modification. Blue bold indicates biosynthetic genes involved in each step. The names in parentheses denote common names.

GSLs are a large group of sulfur-containing secondary metabolites, with more than 120 different GSLs identified in Brassicaceae [22]. GSLs are classified into three groups derived from various amino acids: (1) the aliphatic group, derived from Met, Ala, Leu, Ile or Val; (2) the indolic group, derived from Trp; and (3) the aromatic group, derived from Phe or Tyr [23]. The GSLs of the three groups are synthesized in three stages (Figure 1). The precursor amino acids of the aliphatic and aromatic groups are chain-elongated in the early phase, and then oxime is converted into the core GSL structure in the second stage, respectively. Members of the indolic group are transformed into the core GSL structure from the oxime at the first stage, without undergoing the chain-elongation stage. Finally, the side chains of the core structures are modified by oxidation, elimination, alkylation or esterification in the aliphatic and indolic groups [14]. Aliphatic GSLs are the most abundant, at about 57–97% of the total GSL content, while aromatic GSLs are minor components in *Brassica* species [24,25,26]. The GSLs of the aliphatic and aromatic groups have unique properties for human health and livestock and have roles in oncogenesis, disease, and nutrition [27]. Glucoraphanin, a precursor of sulforaphane, can act as an anticarcinogenic agent in human cells [28]. Sinigrin has been reported in high amounts in black mustard (*B. nigra*), broccoli (*B. oleracea*), and Indian mustard (*B. juncea*), and has been shown to have antioxidant, anticancer, and antifungal effects [29]. Furthermore, phenethyl isothiocyanate (PEITC), which is formed from gluconasturtiin of the aromatic GSL group, has been shown to have a significant chemo-preventative effect against human prostate cancer [30]. Conversely, progoitrin causes goiter disease in mammals, and impedes the use of *Brassica* crops for cattle feed [31]. GSLs of the indolic groups, such as glucobrassicin, 4-hydroxy glucobrassicin, neoglucobrassicin, and 4-methoxy glucobrassicin, have demonstrated that these breakdown products contribute to defense against biological stresses, including pests and pathogens [32,33]. Tryptophan-derived indole-3-acetaldoxime in the indolic group—also known as a precursor for auxin related to plant growth and development—and camalexin biosynthesis controlling deter bacterial and fungal pathogens [34,35]. Recently, some aliphatic and indolic GSLs have been reported that are involved in primary metabolism in plants, such as nitrogen and sulfur sources, and abiotic stresses, such as salinity, light, and elevated CO_2_ [36,37,38,39,40].

### 2.2. Regulators of the GSL Biosynthesis Pathway in the Model Plant Arabidopsis 

Most of the structural genes and transcription factors involved in GSL biosynthesis have been identified from diverse molecular and genetic studies in *Arabidopsis* [18,19,33]. To date, more than 40 structural genes and eight TFs involved in the GSL biosynthesis pathway have been identified in *Arabidopsis*. Dof1.1 for DNA binding with one finger has been reported as the regulator of networks that positively control indolic GSLs in *A. thaliana* [41]. IQD (IQ-domain) 1.1 encodes a novel nuclear-localized protein that positively regulates GSL accumulation, and controls the expression of several GSL pathway genes [42]. A further six TFs belong to the group of MYB TFs that contain a R2R3 DNA-binding domain. MYB28, MYB29, and MYB76 are positive regulators of aliphatic GSLs in *A. thaliana*, and high levels of MYB28 transcription result in the production of a large amounts of aliphatic GSLs. MYB34, MYB51, and MYB122 have been shown to regulate indolic GSL biosynthesis [18,19,43,44]. Additionally, recent studies have shown that the MYB34, MYB51, and MYB122 MYB TFs are involved in the biosynthesis of jasmonate acid (JA), abscisic acid (ABA), ethylene (ET), and salicylic acid (SA), which are involved in plant defense [21]. Additionally, these MYB TFs regulate GSL biosynthesis in cooperation with MYC-bHLH (MYC-like basic helix-loop-helix) TFs, known as signaling components of the jasmonic acid pathway [45]. The R2R3 MYB TFs influence the expression of biosynthetic genes related to GSL biosynthesis in *A. thaliana* [19,46]. *AtMYB28* has been reported to regulate the expression of aliphatic GSL biosynthetic genes, such as *AtBCAT-3*, *AtLeuC1* and *MAM1* [19]. In particular, biosynthetic gene *AOP2* (2-oxoglutarate-dependent dioxygenases) expression was increased the transcript levels of the MYB28 and MYB29 of the GSL pathway, controlling GSL biosynthesis [47]. The TFs MYB34, MYB51, and MYB122 positively regulate the accumulation of indolic GSLs and have the potential to upregulate GSL biosynthetic genes, such as *CYP79Bs* [48]. Moreover, all six MYB TFs from *A. thaliana* was shown to control genes associated with primary sulfate metabolism and are closely related to the GSL biosynthesis network [49].

## 3. MYB TFs Involved in GSL Biosynthesis in Genome-Sequenced *Brassica* Species

### 3.1. Characterization of MYB TFs in Brassica Species 

MYB TFs comprise one of the largest gene families of plant TFs, and play significant roles in the regulation of multiple biological processes, such as developmental and environmental responses, and metabolic pathways. These MYB TFs are classified into three subfamilies depending on the number of DNA-binding domain repeats and are known to regulate various biological processes by modulating the rate of transcription initiation of target genes [50]. The R2R3 MYB subfamily, containing the DNA-binding domain of the helix–loop–helix repeats R2 and R3, is the largest group of the MYB family and regulates plant-specific processes including primary and secondary metabolism [50]. Interestingly, the DNA binding domain of the R2R3 MYB family contains conserved amino acid sequence motifs, despite the divergence of the amino acid sequence downstream and the conserved motifs contributing to functional conservation [51]. Therefore, an understanding of R2R3 MYB TFs in *Brassica* species may help to elucidate the regulation of secondary metabolism by polyploidy-genome evolution in Brassicaceae.

Six MYB TFs belonging to the R2R3 MYB family have been identified as regulators of GSL biosynthesis in *Arabidopsis* [43,52]. These MYB TFs were also identified using the NCBI (National Center for Biotechnology Information) and *Brassica* databases (http://brassicadb.org/brad/) of genome-sequenced *Brassica* species [9,14,20,53]. In total, we identified 63 orthologous copies corresponding to five MYB TFs, except MYB76. To date, MYB76 TFs have not been defined in the genomes of *Brassica* species, although this has been identified as the positive regulator of aliphatic GSLs in *A. thaliana*. A summary of these 63 orthotonus gene sequences of MYB TFs related to GSL biosynthesis in Brassicaceae is shown in Table 1. More than two copies corresponding to each MYB TF were found in the *Brassica* species. These results indicated that genome duplication events have contributed to the expansion of the R2R3 MYB gene family in the *Brassica* species. The *Brassica* genus, initiating with *B. rapa* [7] and ending finally with *B. juncea* [10] has had its complete genome sequence reported. Only 68% of *B. nigra* (Accession YZ12151) and 85% of *B. oleracea* (var *capitata* line 02-12) of the estimated genome size have been sequenced, and there is some lack of completed gene annotation in their genomes [54]. There are some insufficient gene sequence fragments of orthologous *Brassica* genes for which we can only confirm 52 full-length genes.

**Figure 2:**
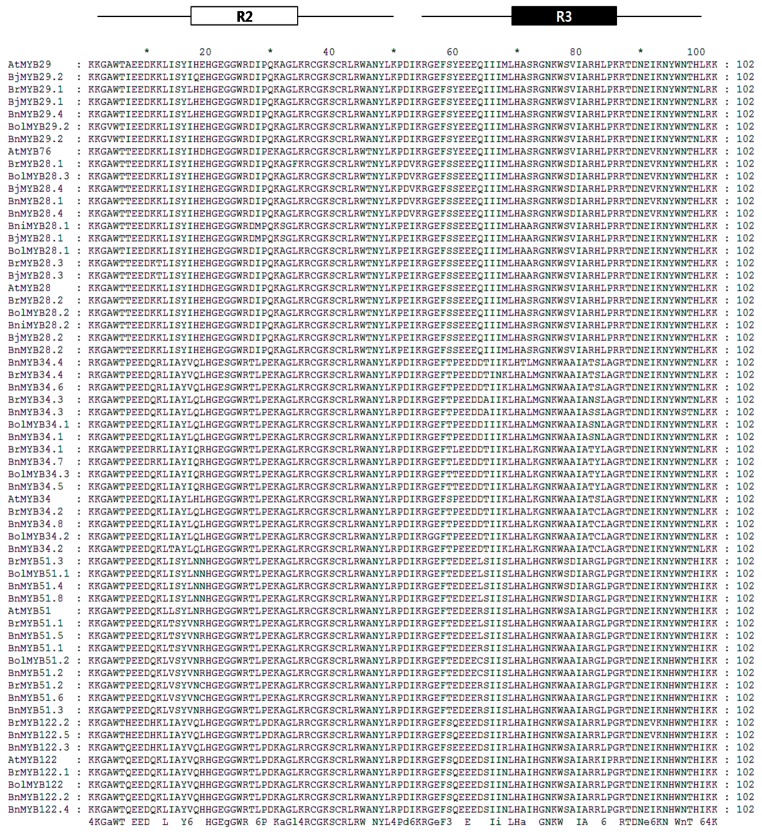
Amino acid sequence alignment of the R2R3 MYB DNA domains in 55 glucosinolate biosynthetic transcription factors in *Brassica* species. The R2 and R3 binding domains are boxed in white and black, respectively. At, *A. thalinala*; Br, *B. rapa*; Bni, *B. nigra*; Bol, *B. oleracea*; Bn, *B. napus*; Bj, *B. juncea*.

**Figure 3:**
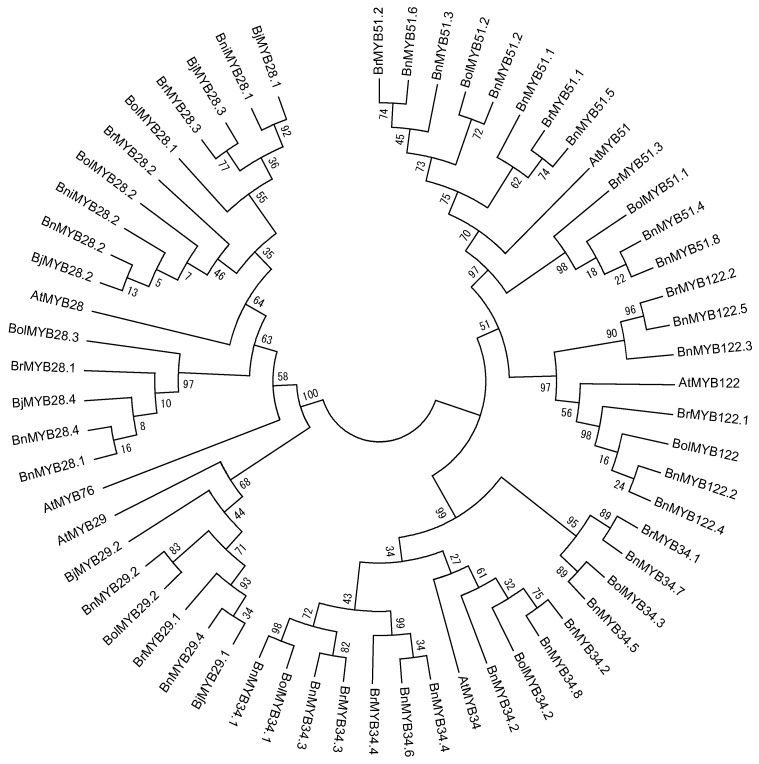
Phylogenetic analysis of R2R3 MYB DNA-binding domains in 55 MYB transcription factors involved in the glucosinolate biosynthesis pathway in *Brassica* species. This tree was constructed using MEGA, version 6, software [58]. Bootstrap values with 1000 replicates are denoted as percentages. At, *A. thalinala*; Br, *B. rapa*; Bni, *B. nigra*; Bol, *B. oleracea*; Bn, *B. napus*; Bj, *B. juncea*.

Although 11 partial sequences of MYB TFs exist in *Brassica*, we were only able to identify the R2R3 MYB DNA-binding domain sequence in *BnMYB28.2*, *BnMYB51.3*, and *BnMYB51.8*. In total, 55 orthologous genes contain a complete R2R3 MYB DNA-binding domain sequence, and these genes showed high conservation at orthologous and paralogous levels (Figure 2). These conserved domains consist of 102 amino acids (AA) and exhibited more than 90% sequence identity with each *A. thaliana*. However, only three motifs (N[R/K/H]VA) were conserved in the 52 full-length MYB TFs of *Brassica* species, despite the 12 motifs conserved in the C-terminal regions of six *AtMYB* TFs. Consequently, variations in gene length (in base pair) and nonsynonymous amino acid sequences are caused by polymorphisms in the downstream C-terminal region. We have identified and reported the conserved R2R3 MYB DNA-binding domains of 13 MYB TFs related to GSL biosynthesis in *B. rapa* [16,24]. A phylogenetic tree was constructed using the amino acid sequences of 61 R2R3 MYB DNA-binding domains containing *A. thaliana* to elucidate evolutionary relationships (Figure 3). Fifty-five MYB TFs were grouped based on six *AtMYB* TFs. Three MYB TFs of *Brassica* related to aliphatic GSLs, including MYB28, 29 and 76, were subgroups, with 76 located between subgroups 28 and 29. MYB34, MY51, and MYB122, involved in indolic GSLs, clustered in a large group, and the MYB34 group formed a subgroup with MYB51. These results indicated that the six MYB TFs related to GSL biosynthesis were evolutionarily conserved in *Brassica* species and exhibit functional conservation. The *AtMYB76* showed a high sequence identity with all MYB29 TFs in the conserved R2R3 MYB DNA-binding domain, but showed a low sequence identity of 69.3% and 62–66% with *AtMYB29* and MYB29 TFs of *Brassica* species in the full length of the MYB29 TFs (no data). Polyploidy can result in chromosomal rearrangements and gene loss, due to unequal rates of sequence evolution of duplicated genes and changes in DNA methylation [55,56,57]. Although the genome assembly of *Brassica* species is not complete, the loss of the MYB76 TF in *Brassica* species may have been caused by genome duplication in evolutionary time. Furthermore, the R2R3 MYB TFs of *B. juncea* and *B. napus*, which have allopolyploid genomes, are closely related to those of *B. rapa*, *B. oleracea*, and *B. nigra*, which have diploid genomes. The amino acid sequence identity of the analyzed R2R3 MYB DNA-binding domains with diploid plants showed the evolutionary origin of the allopolyploid plants in Table 2. Four *BjMYB28* TFs from *B. juncea* (AABB) revealed a high level of sequence similarity to *B. rapa* (AA) and *B. nigra* (BB). *BjMYB28.1* and *BjMYB28.3* were found to possess 100% sequence identity with *BniMYB28.1* and *BrMYB28.3*, respectively, while *BjMYB28.2*, *BrMYB28.2*, and *BniMYB28.2* were fully conserved among the three species. Although *BjMYB28.4* was found to possess a high level of sequence similarity (99.02%) to *BrMYB28.1*, it is possible that an unidentified MYB28 TF of *B. nigra* exists with higher sequence similarity. Similarly, *BnMYB28.1* and *BnMYB28.4* from *B. napus* (AACC) showed 100% sequence similarity with *BolMYB28.3* of *B. oleracea* (CC), and *BnMYB28.2* was found to be 100% conserved in three species. *BnMYB29*s, *BnMYB34*s, *BnMYB51*s, and *BnMYB122*s of *B. napus* were also found to possess high sequence identity with either *BrMYB*s or *BolMYB*s. These results revealed that the *BjMYB* and *BnMYB* TFs originated from the genomes of *BrMYB*s, *BniMYB*s, or *BolMYB*s. Therefore, MYB TFs related to GSL biosynthesis are evolutionary closed and conserved in their R2R3 DNA-binding domains despite duplication and hybridization of two diploid *Brassica* genomes. Furthermore, the number of MYB TFs has increased during evolution, which may have allowed functional diversification and the development of complex networks for the regulation of GSL biosynthesis in polyploidy *Brassica* species.

### 3.2. Functional Description of MYB TFs Related to GSL Biosynthesis in Brassica Species 

Most studies on MYB TFs related to GSL biosynthesis have been performed using *A. thaliana* of the Brassicaceae family as a model plant with a small genome size [21,43,44]. The recent completion of the genome sequencing of *Brassica* species permits the identification of various gene families in its genome. *B. rapa* is a model dicot plant for use in studies of polyploidy-related genome structure and evolution because of the small size of its genome (529 or 485 kb) in the *Brassica* genus [54]. Many putative biosynthetic and regulatory genes related to GSL biosynthesis have been identified in the genome sequence of *B. rapa* [7]. The 13 *BrMYB* TFs that possess a complete coding sequence indicate that paralogous genes arising through gene duplication have led to functional diversity and changes in expression patterns, reflected by genotype-specific variation in *B. rapa* subspecies [24]. The expression of some *BrMYB* TFs, such as *BrMYB28*, *BrMYB34*, and *BrMYB51* also increased under abiotic and biotic stress conditions. Furthermore, functional analysis of three *BrMYB28* TFs has been performed using *Agrobacterium*-mediated transformation in *B. rapa* [16]. The three *BrMYB28* TFs are involved in the regulation of aliphatic, indolic, and aromatic GSL biosynthesis, and in the expression of biosynthetic genes, such as *BrAOP_2_* and *BrGSL-OH* in transgenic *B. rapa*. These results suggested that the regulation of GSL biosynthesis involves a GSL pathway that is more complex than that in *Arabidopsis* due to the complexity of the polyploidy genome in *B. rapa*. Four paralogs of *BjMYB28* in *B. juncea* have been reported as regulators of aliphatic GSL accumulation in transgenic *A. thaliana* and the gene silencing lines of *B. juncea* with a low GSL content [20,59]. Studies on MYB TFs in *B. oleracea* are still not sufficient for the mechanism of *BolMYB* TFs in the regulation of GSLs biosynthesis to be elucidated, although some studies have revealed various expression patterns of biosynthetic genes and *BolMYB* TFs [51,60]. Genetic studies using association mapping have identified multiple loci controlling GSL biosynthesis in *B. napus* and *B. juncea* [61,62]. Recently, the MYB28 TF was identified as the regulator of aliphatic GSL biosynthesis by associative transcriptomics using transcriptome sequencing in *B. napus* [63]. Additionally, association mapping of *B. napus* and *B. juncea* confirmed that MYB28 TFs are associated with GSL content [64,65]. 

Previous studies have demonstrated the role of MYB 28, 29, and 76 as aliphatic GSLs, and MYB 51, 34 and 122 as indolic GSLs, in the regulation of GSL biosynthesis in *A. thaliana*. In the case of *Brassica* species, only a few recent studies have shown that MYB28 TFs positively regulate the accumulation of GSLs. The complexity of the *Brassica* genome, due to an increased number of paralog genes through genome duplication, makes it difficult for a molecular approach to be used to determine the regulation of GSL biosynthesis. In this review, we discussed the role of MYB TFs as important regulators associated with the GSL biosynthesis pathway in *Brassica* species, and provided useful information on the 55 MYB TFs for improved understanding of the regulatory mechanism of GSL biosynthesis in *Brassica* species.

## 4. Conclusions

Recently, six R2R3 MYB TFs controlling the accumulation of various GSLs were reported as regulators of different stress responses and hormones, such as ABA, ethylene, and jasmonate, in *A. thaliana*. Although *Brassica* crops have commercial and scientific value, our understanding of the roles of most MYB TFs is lacking, with the exception of a few MYB TFs related to GSL biosynthesis in *Brassica* species. The 55 R2R3 MYB TFs identified as ortholog genes with *A. thaliana* in *Brassica* species shared a close evolutionary relationship, with a highly conserved DNA-binding amino acid sequence. This will provide valuable information on the mechanisms of MYB TF regulation on unique properties, such as stress responses and various metabolites containing GSL biosynthesis in *Brassica* species with polyploid genomes. Further extensive functional studies of the 55 MYB TFs will help to elucidate the functional diversity of genes via genome duplication in polyploidy plants.

## Figures and Tables

**Table 1:** DNA sequence summary of the MYB transcription factors related to glucosinolate biosynthesis in Brassicaceae.

Species	Gene Name	Gene ID	CDS Length (bp)
*A. thaliana* (diploid)	*AtMYB28*	AT5G61420	1101
	*AtMYB29*	AT5G07690	1011
	*AtMYB34*	AT5G60890	888
	*AtMYB51*	AT1G18570	1059
	*AtMYB76*	AT5G07700	1017
	*AtMYB122*	AT1G74080	1002
*B. rapa* (diploid)	*BrMYB28.1*	Bra012961	1065
	*BrMYB28.2*	Bra035929	1074
	*BrMYB28.3*	Bra029311	1119
	*BrMYB29.1*	Bra005949	993
	*BrMYB29.2*	Bra009245	267 *
	*BrMYB34.1*	Bra035954	909
	*BrMYB34.2*	Bra013000	951
	*BrMYB34.3*	Bra029349	843
	*BrMYB34.4*	Bra029350	930
	*BrMYB51.1*	Bra031035	963
	*BrMYB51.2*	Bra016553	1002
	*BrMYB51.3*	Bra025666	1026
	*BrMYB122.1*	Bra015939	981
	*BrMYB122.2*	Bra008131	1005
*B. oleracea* (diploid)	*BolMYB28.1*	XM_013766140	1059
	*BolMYB28.2*	XM_013750838	1083
	*BolMYB28.3*	XM_013738078	1068
	*BolMYB28.4*	Bol036743	426 *
	*BolMYB29.1*	Bol043899	324 *
	*BolMYB29.2*	XM_013771134	993
	*BolMYB34.1*	Bol007760	843
	*BolMYB34.2*	Bol017062	951
	*BolMYB34.3*	XM_013754685	882
	*BolMYB51.1*	Bol030761	990
	*BolMYB51.2*	Bol013207	1002
	*BolMYB122*	Bol026204	981
*B. nigra* (diploid)	*BniMYB28.1*	JX947841	1053
	*BniMYB28.2*	JX947842	1095
*B. juncea* (allopolyploid)	*BjMYB28.1*	JQ666166	1053
	*BjMYB28.2*	JQ666167	1095
	*BjMYB28.3*	JQ666168	1065
	*BjMYB28.4*	JQ666169	1065
	*BjMYB29.1*	JX316031	993
	*BjMYB29.2*	JX316032	1020
*B. napus* (allopolyploid)	*BnMYB28.1*	GSBRNA2T00040913001	1011
	*BnMYB28.2*	GSBRNA2T00146148001	420 *^,1^
	*BnMYB28.3*	GSBRNA2T00146147001	555 *
	*BnMYB28.4*	GSBRNA2T00113547001	987
	*BnMYB29.1*	GSBRNA2T00092496001	867 *
	*BnMYB29.2*	GSBRNA2T00134508001	966
	*BnMYB29.3*	GSBRNA2T00136149001	1005 *
	*BnMYB29.4*	GSBRNA2T00129660001	948
	*BnMYB34.1*	GSBRNA2T00052239001	843
	*BnMYB34.2*	GSBRNA2T00003879001	948
	*BnMYB34.3*	GSBRNA2T00075530001	843
	*BnMYB34.4*	GSBRNA2T00075529001	918
	*BnMYB34.5*	GSBRNA2T00146117001	882
	*BnMYB34.6*	GSBRNA2T00071741001	867
	*BnMYB34.7*	GSBRNA2T00094719001	909
	*BnMYB34.8*	GSBRNA2T00113599001	951
	*BnMYB51.1*	GSBRNA2T00136837001	963
	*BnMYB51.2*	GSBRNA2T00139596001	1002
	*BnMYB51.3*	GSBRNA2T00139597001	480 *^,1^
	*BnMYB51.4*	GSBRNA2T00006526001	990
	*BnMYB51.5*	GSBRNA2T00016192001	963
	*BnMYB51.6*	GSBRNA2T00102930001	1002
	*BnMYB51.7*	GSBRNA2T00035671001	309 *
	*BnMYB51.8*	GSBRNA2T00035672001	477 *^,1^
	*BnMYB122.1*	GSBRNA2T00088839001	870 *
	*BnMYB122.2*	GSBRNA2T00103485001	1113
	*BnMYB122.3*	GSBRNA2T00082812001	960
	*BnMYB122.4*	GSBRNA2T00102693001	981
	*BnMYB122.5*	GSBRNA2T00092372001	957

* Partial sequence; ^1^ Gene contain R2R3 MYB DNA-binding domain of partial sequence.

**Table 2:** Amino acid sequence identity (%) for the allopolyploidy R2R3 MYB DNA-binding domains of diploidy genes of *Brassica.* Values represent the percentage of sequence similarity and highest percentage demarcated by the gray blocks.

**Allopolyploid**	**Diploid**						
	*BrMYB28.1*	*BrMYB28.2*	*BrMYB28.3*	*BniMYB28.1*	*BniMYB28.2*		
*BjMYB28.1*	92.16	97.06	97.06	100	97.06		
*BjMYB28.2*	95.1	100	98.04	97.06	100		
*BjMYB28.3*	93.14	98.04	100	97.06	98.04		
*BjMYB28.4*	99.02	96.08	94.12	93.14	96.08		
	*BrMYB28.1*	*BrMYB28.2*	*BrMYB28.3*	*BolMYB28.2*	*BolMYB28.3*		
*BnMYB28.1*	99.02	96.08	94.12	96.08	100		
*BnMYB28.2*	95.1	100	98.04	100	96.08		
*BnMYB28.4*	99.02	96.08	94.12	96.08	100		
	*BrMYB29.1*	*BolMYB29.2*					
*BnMYB29.2*	97.06	99.02					
*BnMYB29.4*	100	96.08					
	*BrMYB34.1*	*BrMYB34.2*	*BrMYB34.3*	*BrMYB34.4*	*BolMYB34.1*	*BolMYB34.2*	*BolMYB34.3*
*BnMYB34.1*	90.2	94.12	96.08	91.18	100	92.16	91.18
*BnMYB34.2*	93.14	98.04	93.14	90.2	92.16	98.04	94.12
*BnMYB34.3*	90.2	94.12	98.04	90.2	96.08	92.16	91.18
*BnMYB34.4*	89.22	92.16	91.18	98.04	91.18	90.2	90.2
*BnMYB34.5*	98.04	96.08	92.16	90.2	91.18	94.12	100
*BnMYB34.6*	90.2	93.14	92.16	99.02	92.16	91.18	91.18
*BnMYB34.7*	100	95.1	91.18	89.22	90.2	93.14	98.04
*BnMYB34.8*	95.1	100	95.1	92.16	94.12	98.04	96.08
	*BrMYB51.1*	*BrMYB51.2*	*BrMYB51.3*	*BolMYB51.1*	*BolMYB51.2*		
*BnMYB51.1*	99.02	97.06	94.12	94.12	97.06		
*BnMYB51.2*	97.06	98.04	93.14	93.14	100		
*BnMYB51.3*	97.06	99.02	93.14	93.14	99.02		
*BnMYB51.4*	95.1	93.14	100	100	93.14		
*BnMYB51.5*	100	96.08	95.1	95.1	97.06		
*BnMYB51.6*	96.08	100	93.14	93.14	98.04		
*BnMYB51.8*	95.1	93.14	100	100	93.14		
	*BrMYB122.1*	*BrMYB122.2*	*BolMYB122*				
*BnMYB122.2*	100	94.12	100				
*BnMYB122.3*	95.1	97.06	95.1				
*BnMYB122.4*	100	94.12	100				
*BnMYB122.5*	94.12	100	94.12				
